# Coexistence of Ammonium Transporter and Channel Mechanisms in Amt-Mep-Rh Twin-His Variants Impairs the Filamentation Signaling Capacity of Fungal Mep2 Transceptors

**DOI:** 10.1128/mbio.02913-21

**Published:** 2022-03-01

**Authors:** Gordon Williamson, Ana Sofia Brito, Adriana Bizior, Giulia Tamburrino, Gaëtan Dias Mirandela, Thomas Harris, Paul A. Hoskisson, Ulrich Zachariae, Anna Maria Marini, Mélanie Boeckstaens, Arnaud Javelle

**Affiliations:** a Strathclyde Institute of Pharmacy and Biomedical Sciences, University of Strathclyde, Glasgow, United Kingdom; b Biology of Membrane Transport Laboratory, Department of Molecular Biology, Université Libre de Bruxelles, Gosselies, Belgium; c Computational Biology, School of Life Sciences, University of Dundee, Dundee, United Kingdom; University of Texas Health Science Center

**Keywords:** *Candida albicans*, *Escherichia coli*, *Saccharomyces cerevisiae*, ammonium assimilation, fungal filamentation, secondary transporter mechanism

## Abstract

Ammonium translocation through biological membranes, by the ubiquitous Amt-Mep-Rh family of transporters, plays a key role in all domains of life. Two highly conserved histidine residues protrude into the lumen of the pore of these transporters, forming the family’s characteristic Twin-His motif. It has been hypothesized that the motif is essential to confer the selectivity of the transport mechanism. Here, using a combination of *in vitro* electrophysiology on Escherichia coli AmtB, *in silico* molecular dynamics simulations, and *in vivo* yeast functional complementation assays, we demonstrate that variations in the Twin-His motif trigger a mechanistic switch between a specific transporter, depending on ammonium deprotonation, to an unspecific ion channel activity. We therefore propose that there is no selective filter that governs specificity in Amt-Mep-Rh transporters, but the inherent mechanism of translocation, dependent on the fragmentation of the substrate, ensures the high specificity of the translocation. We show that coexistence of both mechanisms in single Twin-His variants of yeast Mep2 transceptors disrupts the signaling function and so impairs fungal filamentation. These data support a signaling process driven by the transport mechanism of the fungal Mep2 transceptors.

## INTRODUCTION

Cellular ammonium transport is facilitated by the ubiquitous Amt-Mep-Rh superfamily, members of which have been identified in every branch of the tree of life (1). The physiological relevance of Amt-Mep-Rh proteins extends beyond their role in ammonium acquisition as a nitrogen source. In fungi, for instance, in the presence of very low ammonium concentrations, specific Amt-Mep-Rh transporters, the Mep2-like proteins, have been proposed to act as transceptors required for the development of filamentous growth, a dimorphic switch associated with the virulence of pathogenic fungi (2). However, it is currently unclear how the signal that leads to the pseudohyphal growth is initiated by Mep2-like transporters.

Despite the relevance of this protein family, the transport mechanism of Amt-Mep-Rh has remained largely elusive for decades. Crystal structures of various family members revealed a trimeric organization with a narrow conducting transport pore through each monomer lined with hydrophobic residues (3–8). While it was next shown that Amt-Mep-Rh proteins mediate NH_3_ transport after NH_4_^+^ deprotonation (9), other functional studies demonstrated that Archaeoglobus fulgidus Amt1 and 3, Escherichia coli AmtB, and several plant Amts can mediate electrogenic ammonium transport (10–13), questioning the fate of the proton released by NH_4_^+^ fragmentation. We recently showed that two water wires connect the periplasmic side with the cytoplasmic vestibule of EcAmtB (12). These two water wires are interconnected via the H168 residue of the Twin-His, a highly conserved motif constituted of two histidine residues protruding into the lumen of the pore (12, 14). We showed that, after deprotonation of NH_4_^+^ at the periplasmic side, the two interconnected water wires and the Twin-His motif enable H^+^ transfer into the cytoplasm. A parallel pathway, lined by hydrophobic groups within the protein core, facilitates the simultaneous transfer of uncharged NH_3_.

Despite its high level of conservation, the Twin-His motif presents some variations in fungal Amt-Mep-Rh proteins (14–16). Specifically, the first histidine is present in Mep2 transceptors, whereas the Mep1- and Mep3-type proteins feature a natural occupation by a glutamic acid at the first histidine position, defining two functional Amt-Mep-Rh subfamilies in fungi. More recently, using functional characterization in *Xenopus* oocytes, we revealed that Saccharomyces cerevisiae Mep2 mediates electroneutral substrate translocation, while Mep1 performs electrogenic transport, and we proposed that the specific transport mechanism of Mep2 could be responsible for the signal leading to filamentation induction (17). We therefore sought to analyze in more detail the impact of Twin-His variation on the transport mechanism of Amt-Mep-Rh proteins.

Here, we report that altering the Twin-His motif within the pore of E. coli AmtB, S. cerevisiae and Candida albicans Mep2 does not impair ammonium transport function but abolishes the pore selectivity against potassium. We further demonstrate that this loss of selectivity is the result of a mechanistic switch from a transporter-like activity to a channel-like activity governed by a change in the hydrophobicity of the pore. Our findings show that the mechanism of substrate transport ensures the high specificity of the transport. Finally, we show that the mechanistic alteration impacts on the ability of fungal Mep2 proteins to act as transceptors in the development of pseudohyphal growth, supporting the hypothesis that the transport mechanism of Mep2-like proteins is connected to the signal leading to yeast filamentation.

## RESULTS

### Altering pore hydrophobicity does not disrupt transport activity of AmtB.

**FIG 1:**
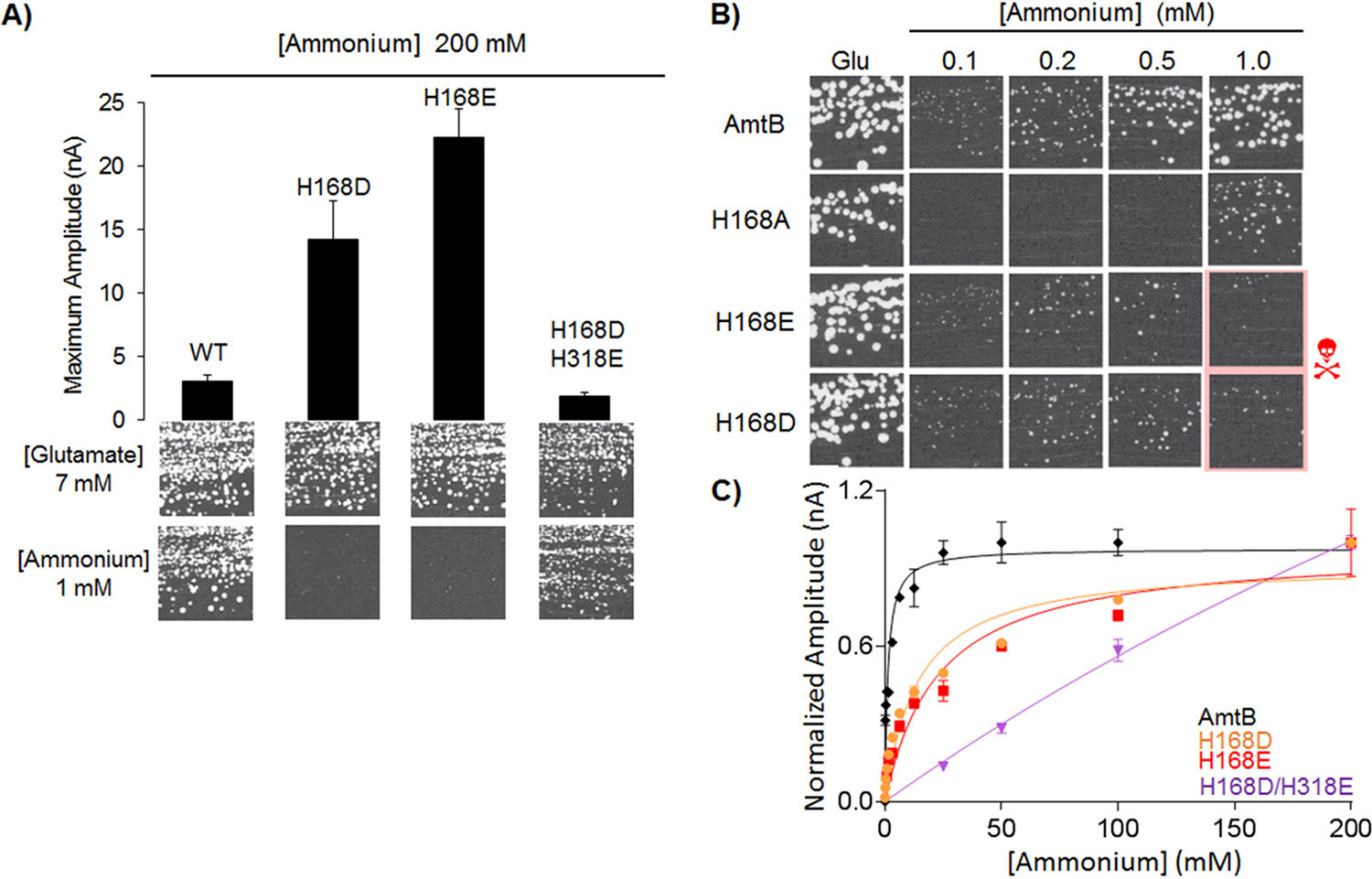
Effect of Twin-His substitution on AmtB ammonium transport activity. (A) (Upper panel) Maximum amplitude of the transient currents measured using SSME following a 200 mM ammonium pulse. (Lower panel) Yeast complementation test after 3 days at 29°C, on potassium glutamate (Glu, positive growth control) or ammonium as the sole nitrogen source. The strain 31019b (*mep1*Δ *mep2*Δ *mep3*Δ *ura3*) was transformed with the pDR195 plasmid, allowing expression of the various AmtB variants. (B) Yeast complementation test, after 5 days at 29°C, in the presence of a range of ammonium concentrations (0.1 mM to 1.0 mM) or glutamate. The red skull shows inhomogenous growth (C) Kinetics analysis for the transport of ammonium using SSME. The maximum amplitudes recorded after a 200 mM ammonium pulse have been normalized to 1.0 for comparison.

To determine the effect of altering the hydrophobicity of the pore on transport activity, we first looked at the effect of single acidic substitutions H168E and H168D within the Twin-His motif of AmtB, mimicking the glutamate substitution present in the fungal Mep1/3-type transporters. We purified and reconstituted both AmtB variants into liposomes and measured their activities *in vitro* using solid supported membrane electrophysiology (SSME) and *in vivo* by yeast complementation assays. In proteoliposomes containing AmtB^H168D^ or AmtB^H168E^, an ammonium pulse of 200 mM elicited very high-current amplitudes of 14.23 nA and 22.28 nA, respectively, compared to 3.38 nA observed for the wild type (WT) (Fig. 1A). To confirm that the transient currents correspond to the translocation of ammonium into the proteoliposomes and not to a simple interaction between ammonium and the protein, we investigated the effect of varying the protein density in the liposomes on the transient current. It is expected that increasing the protein density in the liposomes will prolong the decay time of the current if the current represents a complete transport cycle (18). The lifetime of the currents measured for both variants was dependent on the liposomal lipid:protein ratio (LPR), indicating that the currents indeed account for a full translocation cycle (Table 1). Additionally, we measured an increase of the catalytic constants (*K_m_*) for both variants compared to the WT (Fig. 1C, Table 2).

**TABLE 1:** Decay time constants (s^−1^) of transient currents triggered after an ammonium, methylammonium, or potassium pulse of 200 mM in proteoliposomes containing AmtB variants at various LPR^*a*^

Variant	Ammonium		MeA		Potassium	
	LPR 10	LPR 5	LPR 10	LPR 5	LPR 10	LPR 5
WT	13.4 ± 1.5	18.7 ± 1.0	3.6 ± 0.5	8.3 ± 1.2	NC	NC
H168D	31.4 ± 1.3	38.1 ± 4.1	17.7 ± 2.1	26.4 ± 4.2	NC	NC
H168E	45.5 ± 4.8	53.9 ± 1.6	27.0 ± 1.9	36.4 ± 3.1	NC	NC
H168D/H318E	15.8 ± 1.1	17.8 ± 0.7	10.5 ± 2.5	13.9 ± 1.8	2.3 ± 0.4	4.9 ± 0.2

a NC, no transient current recorded.

**TABLE 2:** *K_m_* (mM) of AmtB variants for ammonium, methylammonium, and potassium, using SSME^*a*^

Variant	Ammonium	MeA	Potassium
WT	0.8 ± 0.1	49.86 ± 4.76	NC
H168D	14.02 ± 4.08	55.09 ± 12.67	NC
H168E	12.87 ± 7.79	86.00 ± 9.46	NC
H168D/H318E	NA	NA	NA

a NC, no transient current recorded; NA, not applicable.

Expressed in a S. cerevisiae triple-*mep*Δ strain deprived of its three endogenous Mep ammonium transporters, both variants were unable to restore growth on 1 mM ammonium after 3 days, suggesting a loss of function (Fig. 1A). However, after 5 days, an inhomogeneous weak growth was observed (Fig. 1B). Previous work has demonstrated that noncontrolled ammonium influx can be toxic to S. cerevisiae (19, 20). To test if this inhomogeneous growth could be linked to toxic ammonium influx, the yeast complementation assay was repeated with a lower range of ammonium concentrations (0.1 to 1.0 mM) (Fig. 1B). The complementation ensured by both AmtB^H168D^ and AmtB^H168E^ variants was improved by decreasing the ammonium concentrations. These results suggest that the acidic substitution at position 168 drastically increases the ammonium flux through AmtB, rendering it toxic to yeast cells. We did not previously observe ammonium toxicity with the AmtB^H168E^ variant expressed in yeast (15). In those experiments, AmtB^H168E^ was expressed under the control of another promoter, *MET25*, repressible by methionine supplementation. The presence of ammonium under its sulfate salt may lead to partial repression of the *MET25* promoter. In the present experiment, the variants are expressed under the control of the *PMA1* promoter, a stronger promoter than *MET25*. Therefore, enhanced expression of the variants in the present experiment could explain the observed ammonium toxicity.

A double H168D/H318E mutation has previously been shown to impact AmtB specificity (21). To further probe the impact of pore hydrophobicity on the activity and specificity of AmtB, we substituted both residues of the Twin-His motif with acidic residues (AmtB^H168D/H318E^). SSME measurements revealed that a 200 mM NH_4_^+^ pulse elicited an LPR-dependent transient current with a maximum amplitude of 1.84 nA in proteoliposomes containing AmtB^H168D/H318E^, a 1.8-fold reduction compared to WT AmtB (Fig. 1A, Table 1). Additionally, a catalytic constant (*K_m_*) for AmtB^H168D/H318E^ could not be determined, as saturation could not be achieved, even following an ammonium pulse of 200 mM (Fig. 1C, Table 2). When expressed in the S. cerevisiae triple-*mep*Δ strain, AmtB^H168D/H318E^ was able to restore cell growth on low ammonium concentrations (Fig. 1A). These data demonstrate that while AmtB^H168D/H318E^ is still functionally active, its activity seems to be reduced compared to WT AmtB. The absence of saturable kinetics further suggests that the variant behaves like a channel rather than having transporter-like activity. We previously observed this type of behavior when we replaced the Twin-His motif with alanine residues, although the mechanism of this switch remained elusive (12).

**FIG 2:**
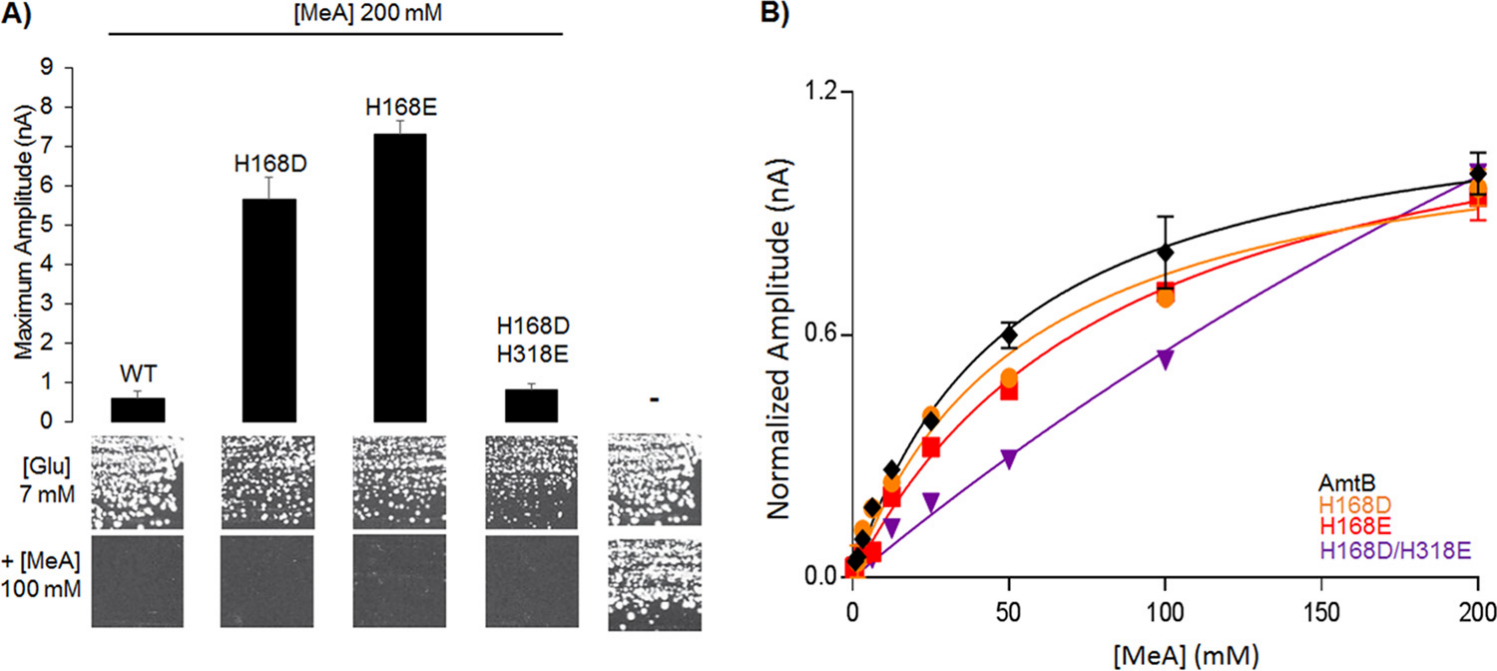
Effect of Twin-His substitution on AmtB methylammonium transport activity. (A) (Upper panel) maximum amplitude of the transient current measured using SSME following a 200 mM methylammonium (MeA) pulse. (Lower panel) Yeast complementation test, after 3 days at 29°C, on solid minimal medium containing, as the sole nitrogen source, potassium glutamate 7 mM (glu, positive growth control) supplemented or not with 100 mM methylammonium. The strain 31019b (*mep1*Δ *mep2*Δ *mep3*Δ *ura3*) was transformed with the empty pDR195 plasmid (–), or with pDR195 allowing expression of the various AmtB variants. (B) Kinetics analysis for the transport of methylammonium using SSME. The maximum amplitudes recorded after a 200 mM MeA pulse have been normalized to 1.0 for comparison.

Previously, methylammonium (MeA) was used as a substrate analogue for ammonium. However, it triggers transient currents of only 15 to 20% of those elicited by ammonium, indicating strong substrate discrimination by AmtB (11, 16, 22). To determine if this level of discrimination is maintained in the Twin-His variants, the AmtB^H168D^ and AmtB^H168E^ variants were subjected to a pulse of 200 mM MeA during SSME. Both AmtB^H168D^ and AmtB^H168E^ exhibited increased activity compared to WT AmtB (9-fold and 12-fold increase in current amplitude, respectively). The double mutant AmtB^H168D/H318E^ showed a maximum amplitude comparable to WT AmtB (Fig. 2A), but it was not possible to determine a catalytic constant, again due to the lack of saturation (Fig. 2B, Table 2). These data indicate that the mutations do not alter the ability of AmtB to discriminate between MeA and ammonium *in vitro*. Yeast complementation was carried out in parallel. MeA cannot be metabolized by yeast cells and is toxic at high external concentrations (15, 23). The native AmtB and all the Twin-His variants allowed growth of triple-*mep*Δ S. cerevisiae cells on glutamate medium, but not when it was supplemented with MeA (Fig. 2A). This shows that all the variants are also active in transporting MeA in yeast.

### Altering pore hydrophobicity abolishes transport selectivity.

**FIG 3:**
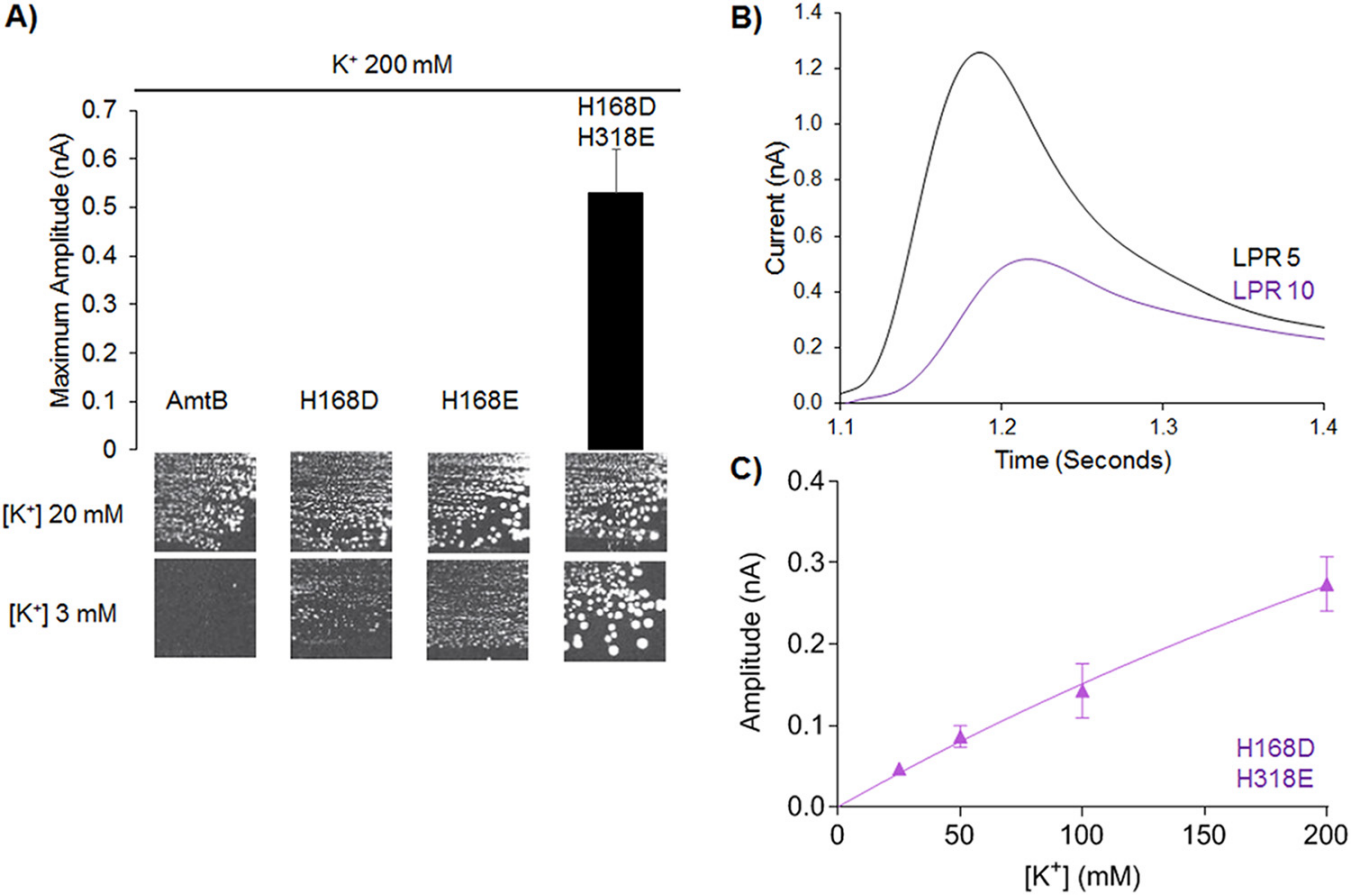
Potassium (K^+^) transport in AmtB Twin-His variants. (A) (Upper panel) Maximum amplitude of the transient current measured using SSME following a 200 mM potassium pulse. (Lower panel) Yeast complementation test, after 5 days at 29°C, in the presence of 20 mM or 3 mM potassium. The #228 strain (*mep1*Δ *mep2*Δ *mep3*Δ *trk1*Δ *trk2*Δ *leu2 ura3*) was transformed with the pDR195 plasmid allowing expression of the various AmtB variants. (B) Transient currents measured using SSME following a 200 mM potassium pulse with AmtB^H168D/H318E^ reconstituted into proteoliposomes at a LPR 5 (black) or 10 (purple). (C) Kinetics analysis for the transport of potassium using SSME.

The findings reported above showed that altering the hydrophobicity of the pore does not inactivate AmtB but has a substantial impact on the transport mechanism. Thus, we next investigated whether the substitutions also affect the ammonium/MeA selectivity of AmtB against competing ions. We focused on K^+^, as it has an ionic radius similar to that of NH_4_^+^. A 200 mM K^+^ pulse failed to elicit a measurable current in AmtB^H168D^ or AmtB^H168E^ proteoliposomes but was able to trigger a clear transient current in AmtB^H168D/H318E^ (Fig. 3A). The amplitude and decay time of the currents recorded for AmtB^H168D/H318E^ are LPR-dependent, confirming that they are caused by K^+^ translocation and are not only the result of protein-substrate interaction (Fig. 3B, Table 1). It was not possible to determine a catalytic constant for AmtB^H168D/H318E^, again due to the lack of saturation, indicating a channel-like rather than a transporter-like activity (Fig. 3C, Table 2). Moreover, all the variants, but not the WT, were able to complement the growth defect of a triple-*mep*Δ *trk*Δ S. cerevisiae strain, which lacks the three endogenous ammonium (Mep) transporters and the 2 major potassium (Trk) transporters, in the presence of a limited concentration of K^+^ (Fig. 3A). The complementation is clearly improved when AmtB^H168D/H318E^ is expressed compared to AmtB^H168D^ or AmtB^H168E^, showing that the single variants are also able to translocate potassium, albeit at a lower rate than AmtB^H168D/H318E^. This could explain why we measured a current after a K^+^ pulse in the proteoliposomes containing AmtB^H168D/H318E^ but not with AmtB^H168D^ or AmtB^H168E^. We reason that there could be an inverse relationship between the hydrophobicity of the central pore and the selectivity of AmtB.

### Loss of AmtB selectivity is due to increased pore hydration accompanied by a mechanistic change.

**FIG 4:**
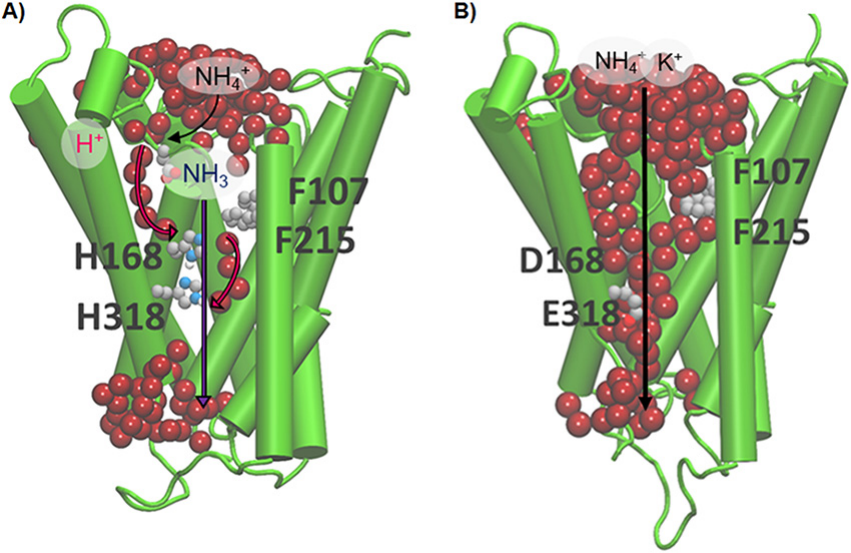
Schematic comparison of transport in WT AmtB and AmtB^H168D/H318E^. (A) Molecular dynamic simulation of the AmtB monomer, showing the interconnected water wires (represented as red spheres). Following sequestration of NH_4_^+^ at the periplasmic face, NH_4_^+^ is deprotonated, before passing the “Phe-Gate,” representing the first hydrophobic barrier, and H^+^ and NH_3_ follow two separate pathways to join the cytoplasm (magenta arrows depict the pathway for H^+^ transfer, dark blue arrows for NH_3_) facilitated by the presence of two internal water wires. (B) Molecular dynamic simulation of the AmtB^H168D/H318E^ monomer, showing the pore filled with water molecules. Due to the increased hydration within the pore, periplasmic NH_4_^+^ and K^+^ are translocated directly through the central pore to the cytoplasm.

To understand the loss of substrate selectivity observed in the AmtB variants and the switch from transporter-like to channel-like activity observed in the SSME recordings with the AmtB^H168D/H318E^ variant, molecular dynamics simulations were conducted. Our previously proposed model for the transport mechanism of AmtB suggests that, after deprotonation of NH_4_^+^ at the periplasmic side, two interconnected water wires enable H^+^ transfer into the cytoplasm. A parallel pathway, lined with hydrophobic groups within the protein core, facilitates the simultaneous transfer of NH_3_ (Fig. 4A) (12).

Molecular dynamics simulations based on alterations of the Twin-His motif by the introduction of charged residues show that this destabilizes and widens the pore, causing it to fill with water and forming a continuous aqueous channel (Fig. 4B). This represents a significant change from the ordered single-file chains of water molecules, separated by the Twin-His motif, observed in the simulations with the WT. The formation of a wide aqueous pore at the expense of discrete water chains disrupts the mechanism of transport and compromises the ability of AmtB to act as a specific transporter, since the newly flooded pore can enable the direct passage of hydrated cations.

**FIG 5:**
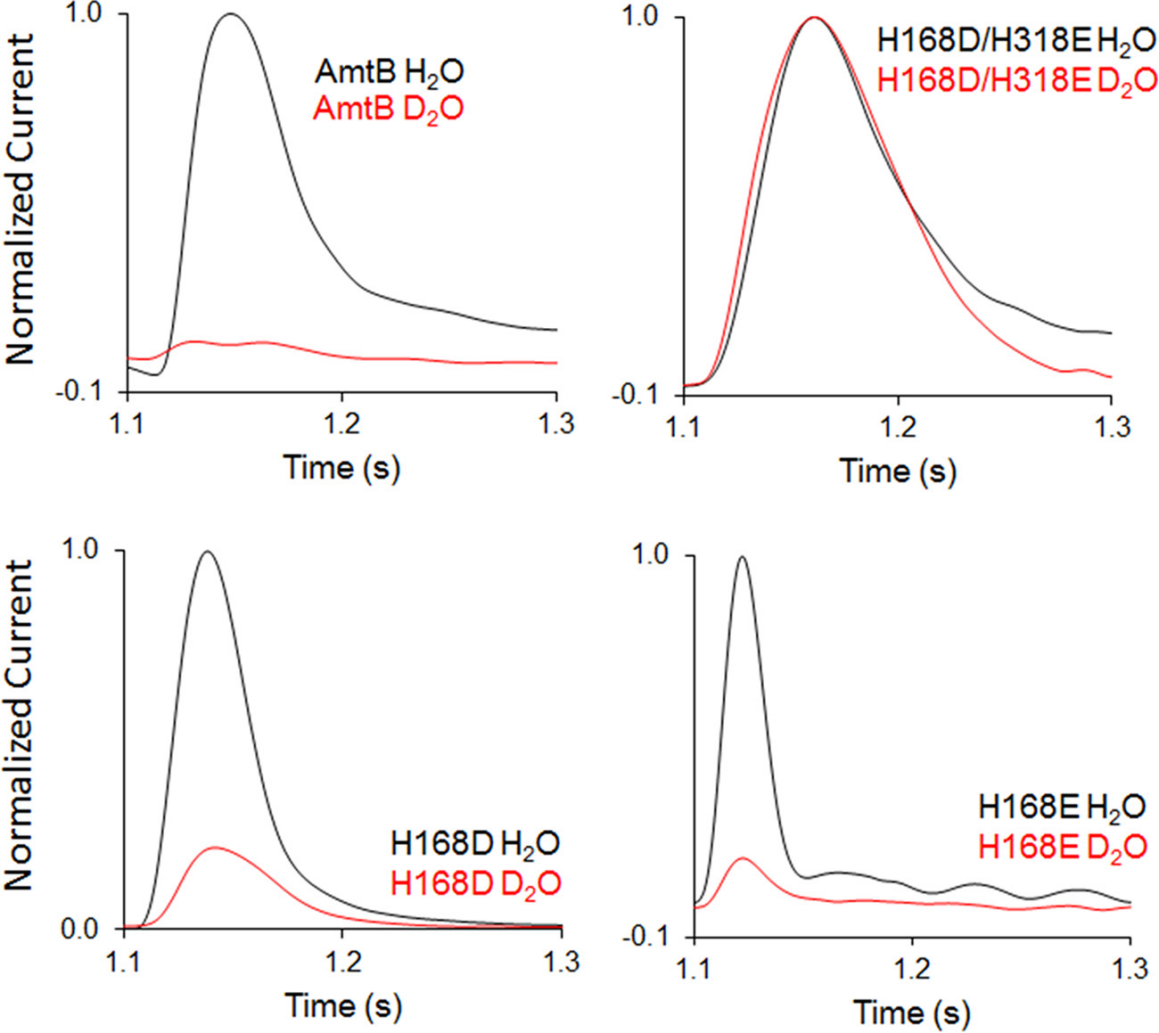
Hydrophobicity of the AmtB transport pore governs mechanistic switch. Transient currents measured using SSME following a 200 mM ammonium pulse on sensors prepared with solutions containing either H_2_O (black) or D_2_O (red) in WT AmtB, AmtB^H168D/H318E^, AmtB^H168D^, or AmtB^H168E^. D_2_O currents have been normalized to respective H_2_O currents.

To test if the single-file water wires are indeed disrupted by the substitutions, we employed a D_2_O-based SSME assay. Because the strength of a covalent bond involving deuterium increases compared to hydrogen, proton mobility is reduced by 30% for each D_2_O molecule compared to H_2_O (24). If the water wires are intact and are required for the transport mechanism, replacement of H_2_O with D*_2_*O is expected to result in the complete abolishment of current when measured by SSME. If, however, the mechanism does not require the water wires, the substitution of H_2_O with D_2_O should not affect the observed current following an ammonium pulse. As previously observed in WT AmtB, no current was measured under D_2_O conditions (Fig. 5) (12). In AmtB^H168D^ and AmtB^H168E^, the current observed following a 200 mM ammonium pulse was diminished by about 4-fold in the presence of D_2_O compared to H_2_O, but not completely abolished (Fig. 5). The stark reduction of current in the presence of D_2_O indicates that proton hopping remains a mechanistic feature within AmtB^H168D^ and AmtB^H168E^. However, the lack of complete abolition implies that some charge translocation is occurring by another mechanism. These data suggest that two transport mechanisms are used by these variants, one depending on NH_4_^+^ deprotonation, and the other allowing the occasional passage of hydrated NH_4_^+^ or K^+^, as shown in Fig. 4. This result is in agreement with the capacity of these variants to transport K^+^ in yeast (Fig. 3A).

In contrast, in AmtB^H168D/H318E^, a 200 mM ammonium pulse elicits transient currents of similar magnitude with either D_2_O or H_2_O (Fig. 5). This indicates that the central mechanism of proton hopping observed in WT AmtB is no longer a mechanistic feature of AmtB^H168D/H318E^ which gains the ability to directly transport NH_4_^+^ without deprotonation. These findings demonstrate that hydrophilic substitutions within the Twin-His motif gradually lead to a switch in the transport mechanism of AmtB protein from transporter to channel-like, which is observed in AmtB^H168D/H318E^. In the latter, NH_4_^+^ is translocated as an intact cation in its hydrated form, abolishing transport specificity (Fig. 4). These data also explain why the ammonium/MeA/potassium transport activity cannot be saturated in proteoliposomes containing AmtB^H168D/H318E^ and why this variant is highly efficient in potassium transport (Fig. 1, 2, and 3, Table 2).

### The Twin-His motif is involved in the transport specificity of ScMep2.

**FIG 6:**
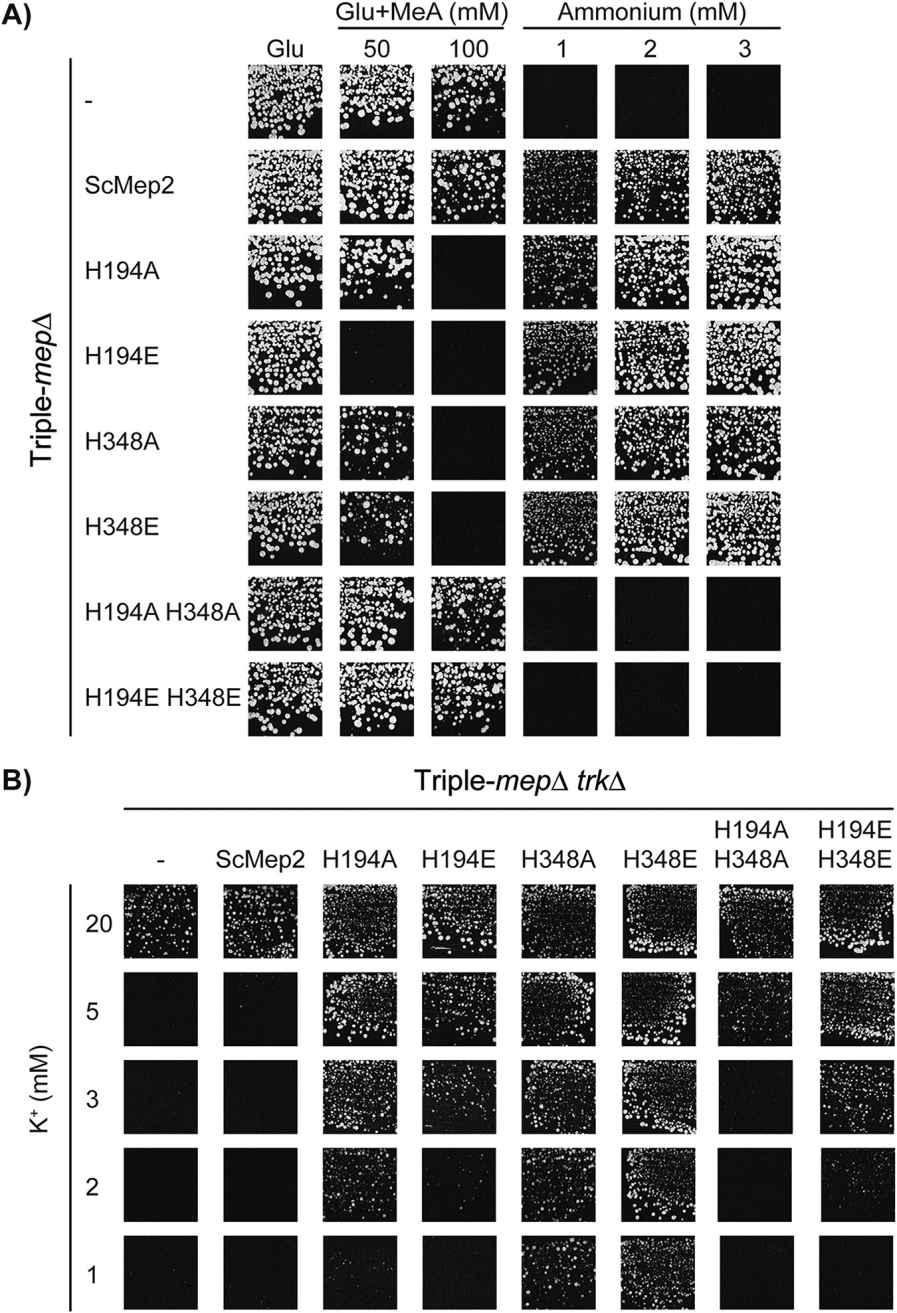
Effect of Twin-His substitutions on ScMep2 transport activity and specificity. (A) Growth tests, after 4 days at 29°C, on solid minimal medium containing, as the sole nitrogen source, ammonium 1, 2, and 3 mM or potassium glutamate (Glu, positive growth control) supplemented or not with 50 or 100 mM methylammonium (MeA). The Saccharomyces cerevisiae strain 31019b (*mep1*Δ *mep2*Δ *mep3*Δ *ura3*) was transformed with the pFL38 empty plasmid (–) or with YCpMep2, YCpMep2^H194A^, YCpMep2^H194E^, YCpMep2^H348A^, YCpMep2^H348E^, YCpMep2^H194A/H348A^, or YCpMep2^H194E/H348E^. (B) Growth tests, after 4 days at 29°C, on solid minimal medium containing different potassium concentrations (from 1 mM to 20 mM) and in the presence of sodium glutamate as the nitrogen source. The Saccharomyces cerevisiae strain #228 (*mep1*Δ *mep2*Δ *mep3*Δ *trk1*Δ *trk2*Δ *leu2 ura3*) was transformed with the pFL38 empty plasmid (–) or with YCpMep2, YCpMep2^H194A^, YCpMep2^H194E^, YCpMep2^H348A^, YCpMep2^H348E^, YCpMep2^H194A/H348A^, or YCpMep2^H194E/H348E^.

Previously, we showed that alanine mutations in the Twin-His motif (AmtB^H168A^ and AmtB^H318A^) also alter the selectivity of the AmtB pore, resulting in the ability of the variants to translocate K^+^ ions (12). To understand the general importance of the hydrophobicity of the central pore in substrate selectivity, we simultaneously compared Twin-His variants of S. cerevisiae Mep2 with single or double mutations in alanine or glutamate residues in terms of ammonium, methylammonium, and potassium transport functionality. We chose a double Twin-His mutation to double E because the paralogue ScMep1 has an E at the His_1_ position and E at the His_2_-1 position. We wished to remain as close as possible to a yeast protein situation known to be functional (ScMep1). We first tested if the S. cerevisiae triple-*mepΔ* strain expressing ScMep2^H194A^, ScMep2^H194E^, ScMep2^H348A^, ScMep2^H348E^, ScMep2^H194A/H348A^, or ScMep2^H194E/H348E^ was able to grow on low ammonium concentration, compared to cells expressing native ScMep2 as the positive control (Fig. 6A). We found that a single substitution of the first or second histidine of the Twin-His motif into alanine or glutamate does not affect in a major way the ammonium transport function of ScMep2 in a home-made buffered medium. However, the ScMep2 double Twin-His mutants (ScMep2^H194A/H348A^ and ScMep2^H194E/H348E^) do not support the growth of the triple-*mepΔ* cells on low ammonium (Fig. 6A). Fluorescence microscopy using Mep2 variants fused to the pHluorin version of green fluorescent protein (GFP) reveals that all variants reach the cell surface, except the double Twin-His variants, which seem to be additionally stacked in the endoplasmic reticulum (Fig. S3A). This could indicate that the failure to complement results from a trafficking problem of the double Twin-His variants.

10.1128/mBio.02913-21.4 FIG S3 Localization of Mep2 Twin-His variants fused to the pHluorin version of GFP. Localization of Mep2-pHluorin variants was observed by fluorescence microscopy in cells grown in the presence of proline (0.1%), as the nitrogen source. (A) Triple-*mep*Δ *ura3* cells (strain 31019b) were transformed with pMep2-pHluorin, pMep2^H194A^-pHluorin, pMep2^H194E^-pHluorin, pMep2^H348A^-pHluorin, pMep2^H348E^-pHluorin, pMep2^H194A,H348A^-pHluorin, or pMep2^H194E,H348E^-pHluorin. (B) Triple-*mep*Δ *ura3* (strain 31019b) and triple-*mep*Δ *trk1*Δ *trk2*Δ *leu2 ura3* (strain #228) cells were transformed with pMep2^H194A,H348A^-pHluorin or pMep2^H194E,H348E^-pHluorin. Scale bar, 5 μm. Download FIG S3, DOCX file, 1.0 MB. Copyright © 2022 Williamson et al. 2022 Williamson et al. https://creativecommons.org/licenses/by/4.0/ This content is distributed under the terms of the Creative Commons Attribution 4.0 International license.

We also tested the capacity of ScMep2 variants to intoxicate cells in the presence of high MeA concentrations. Contrary to ScMep1, native ScMep2 is unable to intoxicate cells in the presence of MeA, proposed to be due to a lower maximal transport rate (25). Expression of ScMep2 variants with single His substitutions reduced growth of the triple-*mepΔ* cells in the presence of MeA, suggesting a higher transport flux through the variants or an altered transport mechanism increasing the sensitivity of the cells to the toxic compound (Fig. 6A) (15). The substitution of both histidines in the Twin-His motif was not accompanied by an increased sensitivity of the cells to MeA, which is in agreement with the absence of ammonium transport by the ScMep2^H194A/H348A^ and ScMep2^H194E/H348E^ variants (Fig. 6A). As for AmtB, the Twin-His substitutions of ScMep2 do not affect ammonium/MeA discrimination.

The capacity of the ScMep2 variants to transport K^+^ was next assessed in the triple-*mepΔ* strain further lacking its endogenous high-affinity potassium transporters Trk1/2 in the presence of limiting concentrations of K^+^ (Fig. 6B). In contrast to native ScMep2, expression of any of the ScMep2 Twin-His variants rescues cell growth in the presence of low K^+^ concentrations, with growth efficiency depending on the variant. Of note, the double Twin-His variants enable K^+^ transport function, even if the pHluorin-fused versions are at least partially blocked in the endoplasmic reticulum (Fig. S3B), thereby suggesting that sufficient proteins reach the plasma membrane to produce this phenotype. These data show that ScMep2 Twin-His variants can translocate K^+^, with variants possessing the H348A or H348E mutation being the most efficient in K^+^ transfer (Fig. 6B). These results indicate that, as for AmtB, the mutations in the twin-His motif of the central pore of ScMep2 alters the transport selectivity.

### Coexistence of different transport mechanisms in ScMep2 variants.

**FIG 7:**
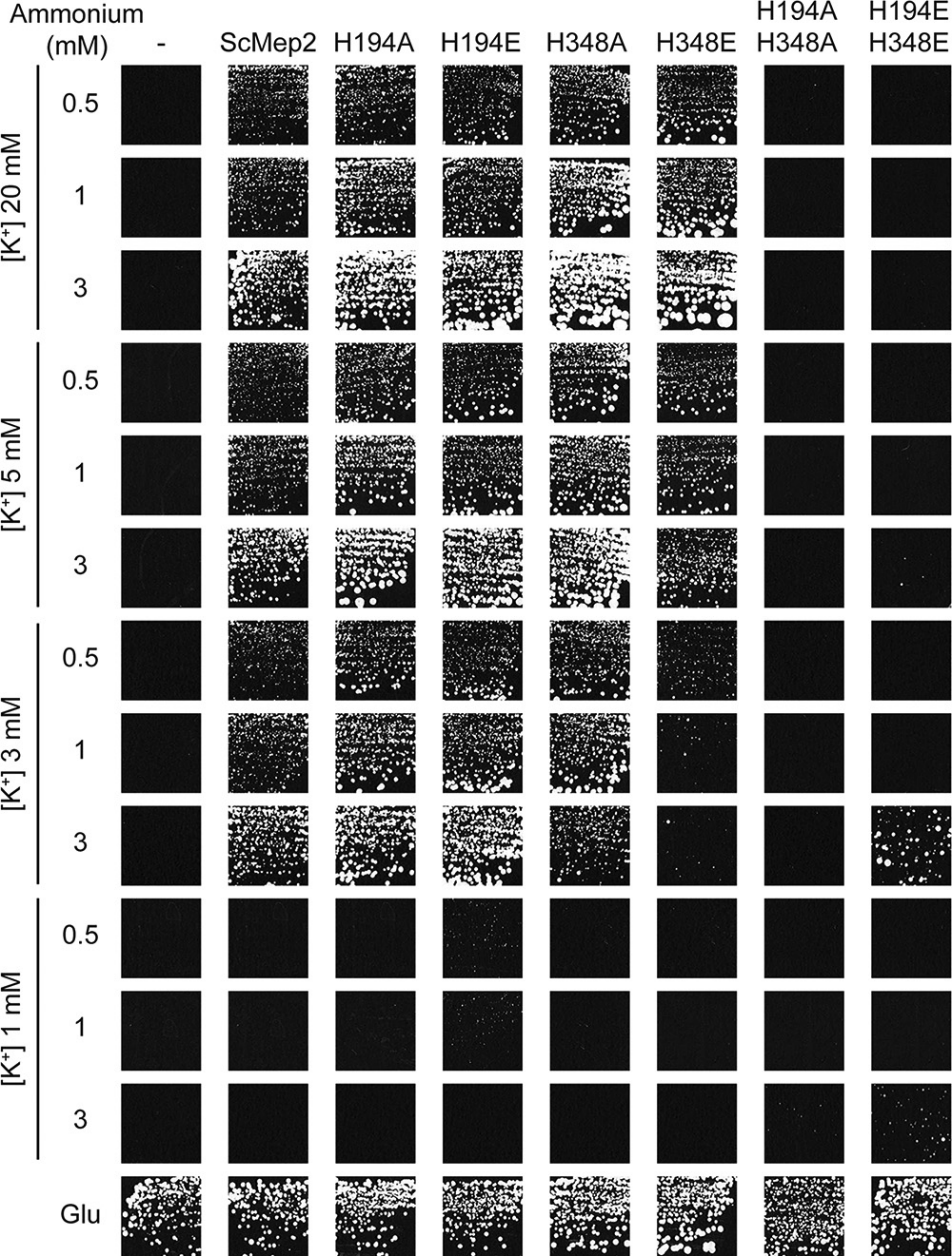
Different transport mechanisms in the ScMep2 Twin-His variants. Growth tests, after 12 days at 29°C, on solid minimal medium containing different potassium concentrations (from 1 mM to 20 mM) and in the presence of different ammonium concentrations (0.5, 1, or 3 mM) as the nitrogen source. The Saccharomyces cerevisiae strain #228 (*mep1*Δ *mep2*Δ *mep3*Δ *trk1*Δ *trk2*Δ *leu2 ura3*) was double transformed with the pFL46 and pFL38 empty plasmids (–) or with pFL46 and YCpMep2, YCpMep2^H194A^, YCpMep2^H194E^, YCpMep2^H348A^, YCpMep2^H348E^, YCpMep2^H194A/H348A^, or YCpMep2^H194E/H348E^. The same solid medium containing 20 mM potassium and potassium glutamate, as the sole nitrogen source, was used as the positive growth control (lower panel; the growth corresponds to day 5 at 29°C).

It is of note that the medium used to test the ammonium transport capacity of the AmtB and ScMep2 variants in yeast contains about 180 mM K^+^. Thus, it appears that single ScMep2 His-variants are able to ensure growth in the presence of 1 mM ammonium despite a very high K^+^ concentration, indicating that potassium does not efficiently compete with ammonium recognition and transport in these variants. This indicates that the single His substitutions allow the coexistence of two transport mechanisms, as proposed for AmtB^H168D^ and AmtB^H168E^. This finding suggests that the first mechanism allows the selective transport of NH_3_ after NH_4_^+^ recognition and deprotonation (as in native Mep2) and is not sensitive to high K^+^. The second mechanism, created by the substitution, could act as an ion channel pathway allowing the passage of K^+^, and likely NH_4_^+^, and would thus be sensitive to competition between both ions. This prompted us to test if the absence of growth under low-ammonium conditions of cells expressing ScMep2^H194A/H348A^ and ScMep2^H194E/H348E^ could be linked to high concentrations of potassium in the medium used. We performed growth tests with the triple-*mepΔ trk*Δ strain in a medium allowing us to simultaneously address the capacity of the variants to transport K^+^ and ammonium. As shown in Fig. 7, ScMep2^H194E/H348E^ allows cell growth in the presence of 3 mM ammonium and equal or lower K^+^ concentrations (1 mM and 3 mM), indicating that the ammonium transport mechanism of this variant is highly sensitive to the potassium concentration. Of note, cells expressing the variant ScMep2^H348E^ appear to grow better when the ammonium concentration decreases specifically in the presence of 3 mM K^+^ and not at higher K^+^ concentrations. One possible explanation could be that upon lowering the concentration of the competitor K^+^ ion, ammonium uptake increases up to toxic levels, as previously observed with AmtB^H168E^ and AmtB^H168D^ variants (Fig. 1B).

Consistent with the conclusions made for AmtB, these data support that simultaneous substitution of the two conserved histidines in ScMep2 results in the formation of a K^+^/NH_4_^+^ channel pathway, while the transport mechanism dependent on NH_4_^+^ deprotonation is abolished. The observation that single histidine variants are able to ensure growth on low ammonium concentrations even in the presence of high potassium concentrations supports the hypothesis that both pathways, one based on ammonium deprotonation and the other linked to direct NH_4_^+^/K^+^ transport, coexist in these variants.

### Influence of the transport mechanism on the capacity of ScMep2 to induce filamentation.

**FIG 8:**
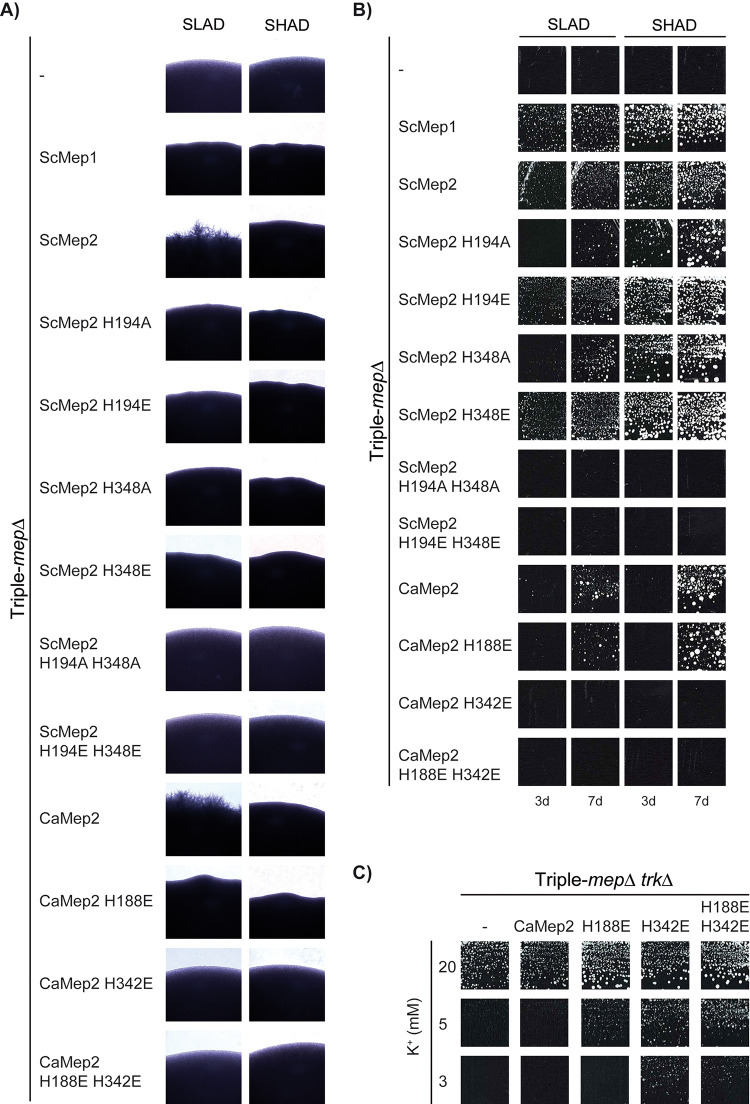
Effect of Twin-His substitutions on the capacity of ScMep2 and CaMep2 to induce fungal filamentation. Homozygous diploid triple-*mep*Δ *ura3* cells (strain ZAM38) were transformed with the pFL38 empty plasmid (–) or with YCpMep2, YCpMep2^H194A^, YCpMep2^H194E^, YCpMep2^H348A^, YCpMep2^H348E^, YCpMep2^H194A/H348A^, YCpMep2^H194E/H348E^, YCpCaMep2, YCpCaMep2^H188E^, YCpCaMep2^H342E^, or YCpCaMep2^H188E/H342E^. (A) Pseudohyphal growth tests of high-density cell suspensions dropped on SLAD and SHAD media at day 7. (B) Growth tests of low-density cell suspensions on SLAD and SHAD media at days 3 (3d) and 7 (7d), at 29°C. (C) Growth tests, after 4 days at 29°C, on solid minimal medium containing different potassium concentrations (from 2 mM to 20 mM) and sodium glutamate as the nitrogen source. The Saccharomyces cerevisiae strain #228 (*mep1*Δ *mep2*Δ *mep3*Δ *trk1*Δ *trk2*Δ *leu2 ura3*) was transformed with the pFL38 empty plasmid (–) or with YCpCaMep2, YCpCaMep2^H188E^, YCpCaMep2^H342E^, or YCpCaMep2^H188E/H342E^.

ScMep2, but not ScMep1 or ScMep3, is required for the dimorphic switch leading to yeast filamentation in the presence of very low ammonium concentrations (2). H194E and H348A mutations of ScMep2 were proposed to affect the filamentation signaling process under very low ammonium conditions (15, 17, 26). Here, we simultaneously compared the whole set of single and double mutants in the Twin-His motif of ScMep2 to assess the effect of the mechanistic switch from a transporter-like to a channel-like activity on the capacity of ScMep2 to induce filamentation on the appropriate synthetic low ammonium dextrose (SLAD) medium (100 μM ammonium) (Fig. 8A). Synthetic high ammonium dextrose (SHAD) medium (1 mM ammonium) was also used to check for potential constitutive induction of filamentation. In parallel, the ammonium transport functionality of the variants was also evaluated by growth tests on these media (Fig. 8B). We show that, contrary to native ScMep2, filamentation is not observed in the presence of any of the His variants (Fig. 8A), as previously observed with ScMep2^H194E^ and ScMep2^H348A^ (15, 17, 26). As the single His substitutions into alanine slightly affect the functionality of ScMep2 on SLAD (Fig. 8B), the conclusion concerning its signaling capacity appears tricky. ScMep2^H194A/H348A^ and ScMep2^H194E/H348E^ are not able to ensure growth on SLAD (Fig. 8B), probably due to the high potassium concentration (30 mM) compared to the reduced ammonium concentration (100 μM) in this medium; therefore, we cannot draw conclusions on their capacity to induce filamentation. ScMep2^H194E^ and ScMep2^H348E^ have lost their signaling capacity, although they seem functional in ammonium transport function, revealing the importance of the pore hydrophobicity and/or the transport mechanism in the signaling process.

**FIG 9:**
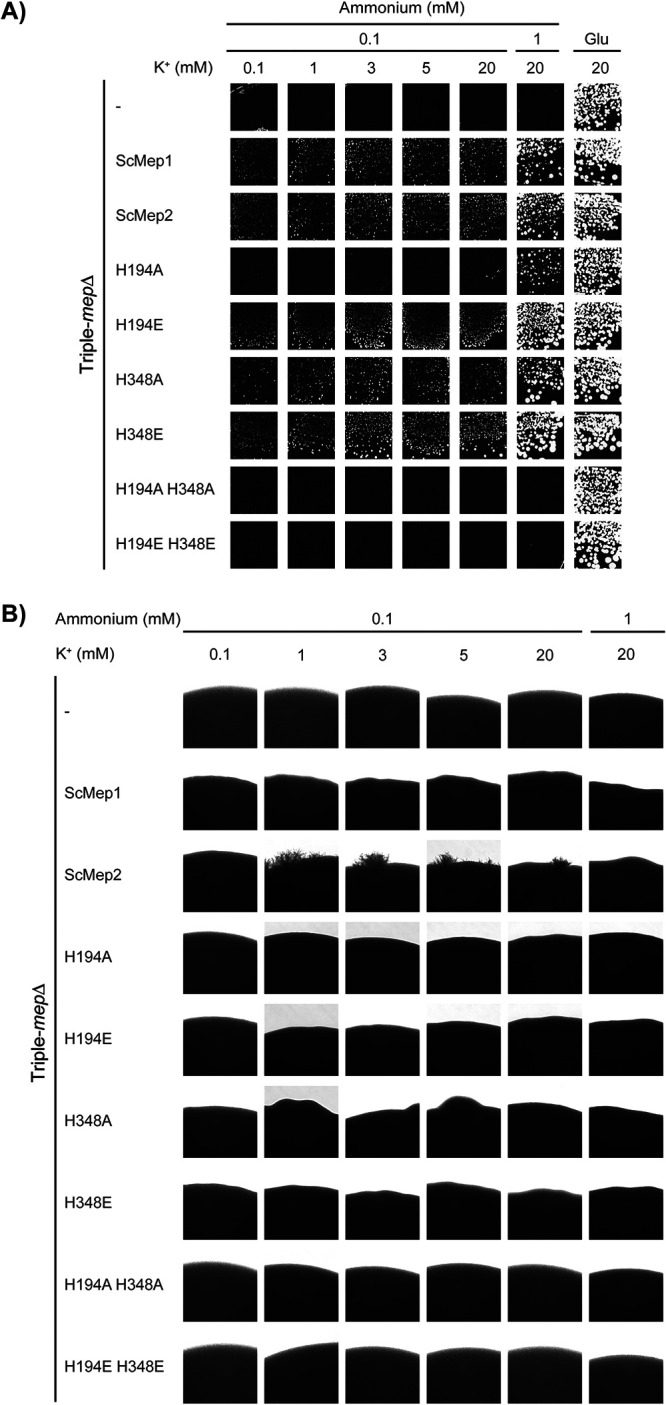
Effect of Twin-His substitutions on the capacity of ScMep2 to induce fungal filamentation at different K^+^ concentrations. Homozygous diploid triple-*mep*Δ *ura3* cells (strain ZAM38) were transformed with the pFL38 empty plasmid (–) or with YCpMep1, YCpMep2, YCpMep2^H194A^, YCpMep2^H194E^, YCpMep2^H348A^, YCpMep2^H348E^, YCpMep2^H194A/H348A^, or YCpMep2^H194E/H348E^. (A) Growth tests, after 7 days at 29°C, on 183 medium containing different potassium concentrations (0.1 mM to 20 mM) and 0.1 mM or 1 mM ammonium, as the nitrogen source. The same medium containing 20 mM potassium and sodium glutamate (Glu), as the sole nitrogen source, was used as the positive growth control. (B) Pseudohyphal growth tests of high-density cell suspensions dropped on 183 medium containing different potassium concentrations (from 0.1 mM to 20 mM) and 0.1 mM or 1 mM ammonium, as the nitrogen source. Cells were incubated 7 days at 29°C.

As potassium could compete with ammonium transport and a high potassium concentration is present in SLAD, we tested the capacity of the Twin-His variants to induce filamentation in a medium containing low potassium concentrations. Filamentation and growth tests were performed in a home-made medium, equivalent to yeast nitrogen base (YNB) medium and allowing control of potassium and ammonium concentrations (0.1 or 1 mM ammonium and a potassium concentration ranging from 0.1 to 20 mM) (Fig. 9A and B). Again, no filamentation is observed with the ScMep2 His variants (Fig. 9B). However, as shown in Fig. 9A, ScMep2^H194A/H348A^ and ScMep2^H194E/H348E^ are not able to sustain growth at 0.1 or 1 mM ammonium, even if the potassium concentration is reduced, suggesting that ammonium transport is strongly inhibited by potassium and is not sufficient to ensure growth. Of note, filamentous growth is not observed with cells expressing ScMep2 at very low potassium concentrations (0.1 mM, Fig. 9B). However, growth is also strongly impaired under this condition, indicating that a minimal potassium concentration is required to ensure optimal growth (Fig. 9A). ScMep2-dependent filamentation is also inhibited by increasing the potassium concentration, which could be due to the inhibition of ammonium translocation by potassium (Fig. 9B). This confirms that the simple presence of ScMep2 at the cell surface is not sufficient for its signaling property; translocation of the substrate is mandatory for signaling (15, 17, 27–29). Taken together, these data suggest that altering the transport mechanism of ScMep2 through Twin-His mutations is correlated with an impaired capacity of the protein to induce filamentation. This result supports the hypothesis that the capacity of ScMep2 to induce filamentation is associated with its substrate translocation mechanism (15, 17).

### Coexistence of different transport mechanisms in CaMep2 variants and influence on filamentation.

CaMep2 from Candida albicans, the orthologue of S. cerevisiae Mep2, is also required to induce filamentation of this human-pathogenic fungus (30). Expression of CaMep2 in S. cerevisiae cells deprived of endogenous ScMep2 restores filamentation, suggesting that a similar mechanism leads to filamentation in both species (17, 30). Here, we compared the impact of a set of Twin-His substitutions in CaMep2 by expressing the variants in S. cerevisiae. As observed with ScMep2, CaMep2 variants mutated in the first and/or second histidine CaMep2^H188E^, CaMep2^H342E^, and CaMep2^H188E/H342E^ are able to translocate K^+^ (Fig. 8C). Growth tests on SLAD and SHAD show that native CaMep2 and CaMep2^H188E^ are less functional in ammonium transport than ScMep2, while CaMep2^H342E^ and CaMep2^H188E/H342E^ are completely nonfunctional, at least in the presence of high potassium concentrations (Fig. 8B). None of the CaMep2 variants are able to allow filamentation (Fig. 8A). Hence, as for ScMep2, the capacity of CaMep2 to induce filamentation also appears to be associated with its transport mechanism.

## DISCUSSION

The data presented show that substitutions within the Twin-His motif of Amt-Mep-Rh decrease the specificity of transport. While this was previously proposed in E. coli, S. cerevisiae, and Arabidopsis thaliana (12, 15, 31), it was assumed that the Twin-His motif was central for the selectivity of the transporters. In this context, the natural occurrence of a glutamic acid in the place of the first histidine in fungal Mep1 and Mep3, but not Mep2 protein, was unclear. Here, our findings lead us to propose a new model that explains the loss of specificity associated with variations in the Twin-His motif. We show that the modification of the Twin-His motif is associated with a change in the pore hydrophobicity increasing its hydration pattern. These findings are supported by our previous X-ray structural analysis, where we showed that the introduction of a charged residue at the Twin-His position drastically enhanced the pore hydration level (14). This increase in hydration governs a switch of the translocation mechanism from a specific transporter, based on substrate fragmentation, to an unspecific channel-like activity, where NH_4_^+^ is transported in its hydrated form and K^+^, an ion of similar size, is able to compete. The mechanism of substrate transport alone ensures the high specificity of the transporter. This is reminiscent of the formate/nitrite transporters that ensure transport selectivity by neutralizing the formate anion by protonation (deprotonation in Amt-Mep-Rh), followed by the passage of the neutral substrate through a lipophilic constriction zone (24). We anticipate that the selective mechanism here evidenced could also apply to Rh members of the Amt-Mep-Rh family, as human RhAG mutations are associated with overhydrated stomatocytosis (OhSt), a hemolytic anemia characterized by the loss of specificity and leakage of important monovalent cations (K^+^, Na^+^) inside red blood cells (32).

In this context, it is important to note that the residues that delineate and stabilize the water wires in AmtB are highly conserved across the whole Amt-Mep-Rh family, which implies that the model proposed for ammonium conduction in AmtB could be conserved among members of the family that exhibit electrogenic activity (12). In line with this hypothesis, our previous results for *Ne*Rh50, a phylogenetically distant homolog of AmtB, demonstrate that its activity is electrogenic (12). In addition, other groups have reported electrogenic activity in other Amt-Mep-Rh members, including Amt1 and Amt3 from Archaeoglobus fulgidus, Amt1;1 from Lycopersicon esculentum, and some human Rh proteins (10, 33, 34). However, electroneutral activity has also been reported for a number of Amt-Mep-Rh proteins (13, 17, 33, 35). Notably, protein isoforms within a single species have been characterized as having different activity. For example, in Arabidopsis thaliana, whose genome encodes 6 *AMT* genes, divided in 2 subfamilies, activity of Amt1 members is electrogenic, while activity of Amt2 is electroneutral (for review see reference 36 and references therein). A similar split is observed in S. cerevisiae and C. albicans Mep2 and Mep1/3 proteins (15, 17). These results suggest that the family possesses more than one unique transport mechanism and, if so, it may be advantageous in order to maintain multiple physiological functions (17). At the moment, the molecular basis underpinning the diversity in transport mechanism in the Amt-Mep-Rh protein family is not fully solved. Our study, however, offers a hint to better apprehend this complex question. We have shown that a single mutation in ScMep2 and AmtB changes a specific transporter to an unspecific ion channel. This is reminiscent of the ubiquitous and very large CLC family of chloride translocators in which apparently similar architecture supports various mechanisms of transport—strict Cl^–^ channel, weak coupled Cl^–^/H^+^ exchanger, strict Cl^–^/H^+^, or NO_3_^–^/H^+^ antiporter (for review see reference 37). Hence, it is now necessary to further investigate the mechanism throughout the Amt-Mep-Rh family, alongside the energetics of the transport and the dynamics of the protein during the transport cycle.

Our results extend beyond the characterization of a new mechanism for selectivity and offer a gateway to better understand signaling mechanisms responsible for fungal filamentation. The mechanism of Mep2-mediated signaling in yeast filamentation remains largely unsolved. Two hypotheses are raised concerning the molecular mechanism of Mep2-mediated signaling. The first one is that Mep2 is a sensor, potentially interacting with signaling partners leading to the induction of filamentation (2). The other one proposes that the specific transport mechanism of Mep2 could underlie the signal leading to filamentation (15, 17). We showed that, in the Mep2 single Twin-His variants, an NH_4_^+^ deprotonation-dependent transport mechanism and a channel-like mechanism (direct NH_4_^+^ transport) coexist. We further showed that this mechanistic alteration impairs the capacity of the S. cerevisiae and C. albicans Mep2 protein to induce filamentation. Altogether, these results support the hypothesis of mechanism-dependent signaling in yeast filamentation. As previously suggested, the difference in the transport mechanism between fungal Mep2 and Mep2^H/E^ could influence in an opposite way the intracellular pH (15, 17). This pH modification could be the signal leading to the dimorphic change. While Mep2 would transport NH_3_ after NH_4_^+^ deprotonation, the current data indicate that the Mep2^H194E^ variant transports NH_4_^+^, thereby leading to acidification. However, we cannot exclude that the mechanism alteration leading to the creation of an ionic channel in single Twin-His Mep2 variants precludes a conformational switch required to transmit the signal to partners. Filamentation is often related to the virulence of pathogenic fungi, such as the human pathogens Candida albicans (38), Histoplasma capsulatum (39), or Cryptococcus neoformans (40). In this context, our results are of particular importance, as the characterization of the conditions regulating the yeast dimorphism may be crucial to better understanding fungal virulence.

## MATERIALS AND METHODS

### Plasmids and mutagenesis.

The plasmids used are listed in Table S1. AmtB mutants were generated using the Quikchange XL site-directed mutagenesis kit (Agilent Technologies), following the manufacturer’s instructions. The *amtB* gene cloned into pET22b(+) was used as the template, as previously described (4).

10.1128/mBio.02913-21.5 TABLE S1 Plasmids used in this study. Download Table S1, DOCX file, 0.03 MB. Copyright © 2022 Williamson et al. 2022 Williamson et al. https://creativecommons.org/licenses/by/4.0/ This content is distributed under the terms of the Creative Commons Attribution 4.0 International license.

Site-directed mutagenesis of *ScMEP2* was performed by GeneCust, using YCpMep2 as the template. The *CaMEP2* (orf19.5672) gene and the mutated *CaMEP2^H188E^* and *CaMEP2^H342E^* genes, with the *ScMEP2* promoter (–661 to −1) and terminator (1 to 262), were synthesized and cloned in pFL38 by GeneCust. Of note, compared to the equivalent plasmids used in reference 17, the *ScMEP2* promoter used in these new plasmids is longer (660 bp compared to 400 bp) and allows a better complementation in growth tests. Plasmid extraction from bacterial cells was performed using the GeneJET plasmid miniprep kit (Thermo Fisher). All constructs were verified by sequencing.

### AmtB purification and solid supported membrane electrophysiology.

AmtB(His)_6_ cloned into the pET22b(+) vector was overexpressed in C43 cells (4, 41), purified (42), and inserted into lipososmes (11) as previously described. The size distribution of proteoliposomes had an average diameter of 110 nm (Fig. S1). The elution profile of all variants and the wild type were identical, showing a single monodisperse peak eluting between 10.4 and 10.6 mL (Fig. S2). Then, 3-mm gold-plated sensors (Nanion Technologies) were prepared (18) and SSME measurements done (12) as previously described. See Text S1 for details.

10.1128/mBio.02913-21.1 TEXT S1 Supplementary information on the methods used for AmtB expression, purification, and insertion into liposomes and solid supported membrane electrophysiology measurements and pseudohyphal growth test. Download Text S1, DOCX file, 0.03 MB. Copyright © 2022 Williamson et al. 2022 Williamson et al. https://creativecommons.org/licenses/by/4.0/ This content is distributed under the terms of the Creative Commons Attribution 4.0 International license.

10.1128/mBio.02913-21.2 FIG S1 Size distribution of the proteoliposomes containing wild-type and variants of AmtB. Dynamic light scattering was used to determine the number-weighted distribution of liposome sizes in the detection reagent. The distribution was monodisperse, with a mean diameter of 110 nm. Download FIG S1, DOCX file, 2.1 MB. Copyright © 2022 Williamson et al. 2022 Williamson et al. https://creativecommons.org/licenses/by/4.0/ This content is distributed under the terms of the Creative Commons Attribution 4.0 International license.

10.1128/mBio.02913-21.3 FIG S2 Size exclusion chromatography analysis of AmtB variants. Gel filtration trace (Superdex 200 10/300 increase) of wild-type AmtB and variants after solubilization of the proteoliposome in 2% *n*-dodecyl-β-d-maltoside (DDM). All of the AmtB variants elute at ∼11.5 mL. Download FIG S2, DOCX file, 1.1 MB. Copyright © 2022 Williamson et al. 2022 Williamson et al. https://creativecommons.org/licenses/by/4.0/ This content is distributed under the terms of the Creative Commons Attribution 4.0 International license.

### Yeast strains and growth conditions.

The S. cerevisiae strains used in this study are the 31019b strain (*mep1Δ mep2Δ mep3Δ ura3*) (43), the #228 strain (*mep1Δ mep2Δ mep3Δ trk1Δ trk2Δ leu2 ura3*) (44), and the ZAM38 strain (*mep1*Δ/*mep1*Δ *mep2*Δ/*mep2*Δ *mep3*Δ/*mep3*Δ *ura3*/*ura3*) (29). Cells were grown at 29°C. Cell transformation was performed as described previously (45).

For growth tests on limiting ammonium concentrations, yeast cells were grown on minimal buffered (pH 6.1) medium (167) containing potassium salts (46). For growth tests on limiting potassium concentrations, a similar minimal buffered (pH 6.1) medium (173) where potassium salts were replaced by sodium salts was used, and KCl was added at the specified concentration; 3% glucose was used as the carbon source. Nitrogen sources were added as required by the experiment and as specified in the text. The nitrogen sources used were 0.1% potassium glutamate, 0.1% sodium glutamate, or (NH_4_)_2_SO_4_ at the specified concentrations referring to the ammonium moiety.

Pseudohyphal growth tests were performed as previously described (47). A suspension of diploid cells was patched onto synthetic low ammonium dextrose (SLAD) and synthetic high ammonium dextrose (SHAD) [0.68% yeast nitrogen base without amino acids and without (NH_4_)_2_SO_4_, containing 3% glucose and 1% bacteriological agar (Oxoid)], respectively, supplemented with 50 μM or 0.5 mM (NH_4_)_2_SO_4_. Pseudohyphal and growth tests on limiting potassium concentrations were performed on a home-made medium (183) equivalent to yeast nitrogen base medium without amino acids, (NH_4_)_2_SO_4_ and potassium salts, and containing low NaH_2_PO_4_ concentrations. (NH_4_)_2_SO_4_ and KCl were added as required by the experiment and as specified in the text. For growth tests, diploid cells were streaked on SLAD, SHAD, and 183 medium to follow the formation of colonies.

All growth experiments were repeated at least twice.

### Molecular dynamics simulations.

The AmtB trimer (PDB code 1U7G) (3) was processed using the CHARMM-GUI web server (48). Any mutations inserted during the crystallization process were reverted to the wild-type form. The studied mutations were introduced into the protein using PyMOL. The N termini and C termini of the subunits were capped with acetyl and N-methyl amide moieties, respectively. The protein was then inserted into a membrane patch of *xy*-dimensions 13 by 13 nm. Unless otherwise specified, a membrane composition of palmitoyl oleoyl phosphatidyl ethanolamine and palmitoyl oleoyl phosphatidyl glycine (POPE/POPG) at a 3:1 ratio was used in order to approximate the composition of a bacterial cytoplasmic membrane. We employed the CHARMM36 forcefield for the protein and counter ions (49). The water molecules were modeled with the TIP3P model (50). Water bonds and distances were constrained by the Settle method (51), and bonds involving hydrogen by the LINCS method (52). In simulations without ammonium, K^+^ and Cl^–^ ions were added to neutralize the system and obtain a bulk ionic concentration of 250 mM. In simulations with ammonium, K^+^ was replaced by NH_4_^+^.

After a steepest-descent energy minimization, the system was equilibrated by six consecutive equilibration steps using position restraints on heavy atoms of 1,000 kJ/mol.nm^2^. The first three equilibration steps were conducted in a constant number, volume, and temperature (NVT) ensemble, applying a Berendsen thermostat (53) to keep the temperature at 310 K. The subsequent steps were conducted under an constant number, pressure, and temperature (NPT) ensemble, using a Berendsen barostat (53) to keep the pressure at 1 bar. Production molecular dynamics simulations were carried out using a v-rescale thermostat (54) with a time constant of 0.2 ps and a Berendsen barostat with semi-isotropic coupling. A timestep of 2 fs was used throughout the simulations.

10.1128/mBio.02913-21.6 TABLE S2 Solid-supported membrane electrophysiology solutions. Download Table S2, DOCX file, 0.02 MB. Copyright © 2022 Williamson et al. 2022 Williamson et al. https://creativecommons.org/licenses/by/4.0/ This content is distributed under the terms of the Creative Commons Attribution 4.0 International license.
