# Supplementary material for: Coexistence of Ammonium Transporter and Channel Mechanisms in Amt-Mep-Rh Twin-His Variants Impairs the Filamentation Signaling Capacity of Fungal Mep2 Transceptors
Source: mBio. 2022 Mar 1;13(2):e02913-21. doi: 10.1128/mbio.02913-21 (PMC9040831; doi:10.1128/mbio.02913-21)
Supplement: FIG S3 [file mbio.02913-21-sf003.docx]

**A)**


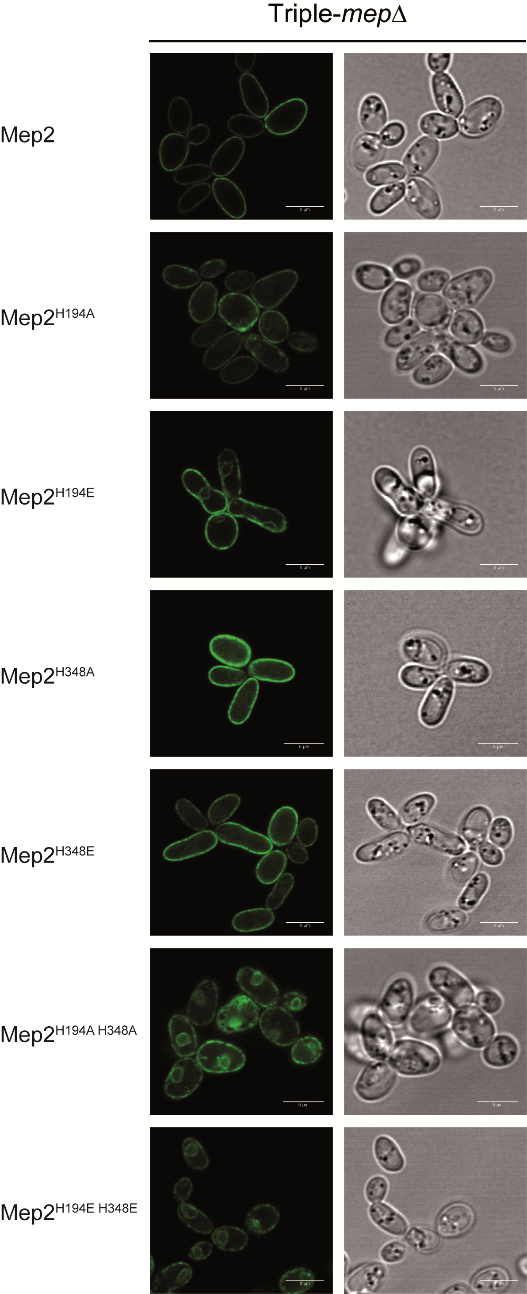


**B)**


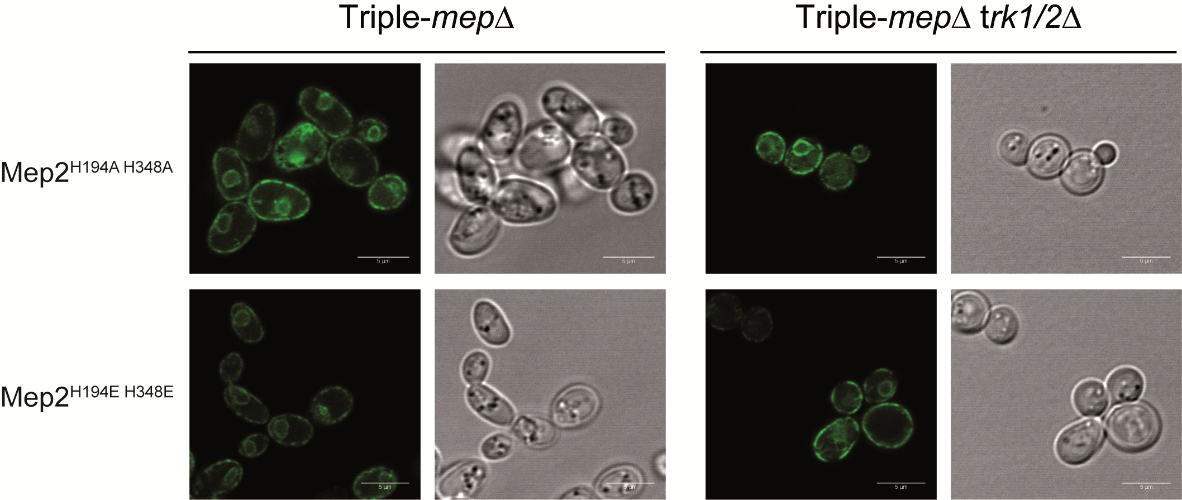


**Figure S3.** **Localization of Mep2 twin-His variants fused to the pHluorin version of GFP.** Localization of Mep2-pHluorin variants was observed by fluorescence microscopy in cells grown in the presence of proline (0.1%), as nitrogen source. (A) Triple-*mepΔ* *ura3* cells (strain 31019b) were transformed with pMep2-pHluorin, pMep2^H194A^-pHluorin, pMep2^H194E^-pHluorin, pMep2^H348A^-pHluorin pMep2^H348E^-pHluorin, pMep2^H194A,H348A^-pHluorin, or pMep2^H194E,H348E^-pHluorin. (B) Triple-*mepΔ ura3* (strain 31019b) and triple-*mepΔ trk1Δ trk2Δ leu2 ura3* (strain #228) cells were transformed with pMep2^H194A,H348A^-pHluorin, or pMep2^H194E,H348E^-pHluorin. Scale bar, 5 μm.
