# Supplementary material for: Coexistence of Ammonium Transporter and Channel Mechanisms in Amt-Mep-Rh Twin-His Variants Impairs the Filamentation Signaling Capacity of Fungal Mep2 Transceptors
Source: mBio. 2022 Mar 1;13(2):e02913-21. doi: 10.1128/mbio.02913-21 (PMC9040831; doi:10.1128/mbio.02913-21)
Supplement: TABLE S1 [file mbio.02913-21-st001.docx]

**Table S1: Plasmids used in this study**

| **Plasmid** | **Description** | **Reference** |
| --- | --- | --- |
| ***E. coli*** |  |  |
| pET22b (+) | High copy bacterial expression vector | Novagen |
| pZheng | pET22b-AmtB(His)_6_ | (1) |
| pAJ2039 | pET22b-AmtB(His)_6_^H168D^ | (2) |
| pGW1 | pET22b-AmtB(His)_6_^H168E^ | This study |
| pGW2 | pET22b-AmtB^H168D/H318E^ | This study |
| ***S. cerevisiae*** |  |  |
| pFL38 | *CEN-ARS URA3* | (3) |
| pFL46 | *2µ LEU2* | (3) |
| pDR195 | *2μ URA3* | (4) |
| pGW6 | pDR195*AMTB^H168E^* | This study |
| pGDM8 | pDR195 *AMTB^H168D^* | This study |
| pGDM9 | pDR195-*AMTB^H168D/H318E^* | This study |
| YCpMep2 | YCpFL38 *MEP2* | (5) |
| YCpMep1 | YCpFL38 *MEP1* | (6) |
| YCpMep2^H194E^ | YCpFL38 *MEP2^H194E^* | (7) |
| YCpMep2^H194A^ | YCpFL38 *MEP2^H194A^* | This study |
| YCpMep2^H348E^ | YCpFL38 *MEP2^H348E^* | This study |
| YCpMep2^H348A^ | YCpFL38 *MEP2^H348A^* | (8) |
| YCpMep2^H194A, H348A^ | YCpFL38 *MEP2^H194A, H348A^* | This study |
| YCpMep2^H194E, H348E^ | YCpFL38 *MEP2^H194E, H348E^* | This study |
| YCpCaMep2 | YCpFL38 PROM *ScMEP2*-*CaMEP2*-TERM *ScMEP2* | This study |
| YCpCaMep2^H188E^ | YCpFL38 PROM *ScMEP2-CaMEP2^H188E^*-TERM *ScMEP2* | This study |
| YCpCaMep2^H342E^ | YCpFL38 PROM *ScMEP2-CaMEP2^H348E^*-TERM *ScMEP2* | This study |
| YCpCaMep2^H188E,H342E^ | YCpFL38 PROM *ScMEP2-CaMEP2^H188E, H348E^*-TERM *ScMEP2* | This study |
| pMep2-pHluorin | p416 PROM Sc*MEP2*-Sc*MEP2* (GA)_5_-pHluorin | (9) |
| pMep2^H194A^-pHluorin | p416 PROM Sc*MEP2*-Sc*MEP2^H194A^* (GA)_5_-pHluorin | This study |
| pMep2^H194E^-pHluorin | p416 PROM Sc*MEP2*-Sc*MEP2^H194E^* (GA)_5_-pHluorin | (9) |
| pMep2^H348A^-pHluorin | p416 PROM Sc*MEP2*-Sc*MEP2^H348A^* (GA)_5_-pHluorin | This study |
| pMep2^H348E^-pHluorin | p416 PROM Sc*MEP2*-Sc*MEP2^H348E^* (GA)_5_-pHluorin | This study |
| pMep2^H194A,H348A^-pHluorin | p416 PROM Sc*MEP2*-Sc*MEP2^H194A,H348A^* (GA)_5_-pHluorin | This study |
| pMep2^H194E,H348E^-pHluorin | p416 PROM Sc*MEP2*-Sc*MEP2^H194E,H348E^* (GA)_5_-pHluorin | This study |

**References**

1. Zheng L, Kostrewa D, BernŠche S, Winkler FK, Li XD. 2004. The mechanism of ammonia transport based on the crystal structure of AmtB of  *E. coli* Proc Natl Acad Sci USA 101:17090-17095.

2. Javelle A, Lupo D, Zheng L, Li XD, Winkler FK, Merrick M. 2006. An unusual twin-his arrangement in the pore of ammonia channels is essential for substrate conductance. Journal of Biological Chemistry 281:39492-39498.

3. Bonneaud N, Ozier-Kalogeropoulos O, Li GY, Labouesse M, Minvielle-Sebastia L, Lacroute F. 1991. A family of low and high copy replicative, integrative and single-stranded *S. cerevisiae/E. coli* shuttle vectors. Yeast 7:609-15.

4. Rentsch D, Laloi M, Rouhara I, Schmelzer E, Delrot S, Frommer WB. 1995. NTR1 encodes a high affinity oligopeptide transporter in Arabidopsis. FEBS Letters 370:264-268.

5. Marini AM, Soussi-Boudekou S, Vissers S, Andre B. 1997. A family of ammonium transporters in *Saccharomyces cerevisae*. Molecular and Cellular Biology 17:4282-4293.

6. Marini AM, Vissers S, Urrestarazu A, Andre B. 1994. Cloning and expression of the MEP1 gene encoding an ammonium transporter in *Saccharomyces cerevisiae.* The EMBO Journal 13:3456-3463.

7. Boeckstaens M, Andre B, Marini AM. 2008. Distinct transport mechanisms in yeast ammonium transport/sensor proteins of the Mep/Amt/Rh family and impact on filamentation. Journal of Biological Chemistry 283:21362-70.

8. Boeckstaens M, Llinares E, Van Vooren P, Marini AM. 2014. The TORC1 effector kinase Npr1 fine tunes the inherent activity of the Mep2 ammonium transport protein. Nature Communication 5:3101.

9. Brito AS, Neuhauser B, Wintjens R, Marini AM, Boeckstaens M. 2020. Yeast filamentation signaling is connected to a specific substrate translocation mechanism of the Mep2 transceptor. PLoS Genetic 16:e1008634.
