# Supplementary material for: Coexistence of Ammonium Transporter and Channel Mechanisms in Amt-Mep-Rh Twin-His Variants Impairs the Filamentation Signaling Capacity of Fungal Mep2 Transceptors
Source: mBio. 2022 Mar 1;13(2):e02913-21. doi: 10.1128/mbio.02913-21 (PMC9040831; doi:10.1128/mbio.02913-21)
Supplement: TEXT S1 [file mbio.02913-21-s0001.docx]

**Coexistence of ammonium transporter and channel mechanisms in Amt-Mep-Rh Twin-His variants impairs the filamentation signalling capacity of fungal Mep2 transceptors**

Gordon Williamson, Ana Sofia Brito, Adriana Bizior, Giulia Tamburrino, Gaëtan Dias Mirandela, Thomas Harris, Paul A. Hoskisson, Ulrich Zachariae, Anna Maria Marini, Mélanie Boeckstaens_,_ and Arnaud Javelle

**Supplementary information**

***AmtB expression and purification***

AmtB(His)_6_ cloned into the pET22b(+) vector was overexpressed in the C43 (1) strain of *E. coli*, as previously describe (2), with minor modifications. 0.03% of *n*-dodecyl-β-d-maltoside (DDM) was used instead of 0.09% *N*,*N*-dimethyldodecylamine-*N*-oxide (LDAO) in the immobilised metal affinity chromatography (IMAC) and size-exclusion chromatography (SEC) buffers. AmtB was kept in the SEC buffer at 4°C prior to insertion into proteoliposomes. All the plamids used in this study are listed Table S1. All constructs were verified by sequencing.

***Insertion of AmtB into liposomes***

AmtB variants were inserted into liposomes containing *E. coli* polar lipids/phosphatidylcholine (POPC) 2/1(wt/wt) as previously described (3). For each AmtB variant, proteoliposomes were prepared at lipid-to-protein ratios (LPRs) of 5, 10, and 50 (wt/wt). The size distribution of proteoliposomes was measured by dynamic light scattering (DLS) using a Zetasizer Nano ZS (Malvern Instruments). This analysis showed that the proteoliposomes had an average diameter of 110 nm (Figure S1). Proteoliposomes were divided into 100 µL aliquots and stored at −80°C.

To ensure that all AmtB variants were correctly inserted into the proteoliposomes, the proteoliposomes were solubilized in 2% DDM and the proteins analyzed by size exclusion chromatography using a superdex 200 (10 × 300) enhanced column. The elution profile of all variants and the wild-type were identical, showing a single monodisperse peak eluting between 10.4–10.6 ml (Figure S2). This demonstrated that all proteins were correctly folded, as trimers, in the proteoliposome

***Solid supported membrane electrophysiology***

3 mm gold plated sensors (Nanion Technologies) were prepared according to the manufacturer’s instructions, as described previously (4). Proteoliposomes/empty liposomes were defrosted and sonicated in a sonication bath at 35 W for 10 seconds and diluted 10-fold in non-activating (NA) solution (Table S2), and 10 µL was added to the surface of the solid-supported membrane (SSM) on the sensor. Sensors were centrifuged at 2500 *g* for 30 minutes and stored at 4°C for a maximum of 48 hours before electrophysiological measurements. For D_2_O experiments, all the solutions were prepared using D_2_O instead of water.

All measurements were made at room temperature (21⁰C) using a SURFE^2^R N1 apparatus (Nanion Technologies) with default parameters (4). Unless otherwise stated, all measurements were carried out using pH 7 buffers. Prior to any measurements, the quality of the sensors was determined by measuring capacitance (15-30 nF) and conductance (<5 nS) and comparing to reference values provided by the manufacturer.

For functional measurements at a fixed pH, a single solution exchange protocol was used with each phase lasting 1 s (4). First, non-activating (NA) solution was injected onto the sensor, followed by activating (A) solution containing the substrate at the desired concentration and finally NA solution (Table S2).

Kinetic parameters were calculated using Graphpad Prism 6 and fitted according to the Michaelis-Menten equation. The decay time of the transient current was calculated by fitting the raw transient current between the apex of the peak and the baseline (after transport) with a non-linear regression using OriginPro 2017 (OriginLab). The regression was done using a one-phase exponential decay function with time constant parameter (equation below) and fit using the Levenberg Marquardt iteration algorithm.

$$y=y_{0}+ A_{1}e^{-x/t_{1}}$$

Where *x* and *y* represent coordinates on the respective axis, *y_0_* represents the offset at a given point, *A* represents the amplitude, and *t* is the time constant.

***Pseudohyphal growth***

*Pseudohyphal growth* tests were performed as previously described (5). A suspension of diploid cells was patched onto Synthetic Low Ammonium Dextrose (SLAD) and Synthetic High Ammonium Dextrose (SHAD) (0.68 % Yeast Nitrogen Base without amino acids and without (NH_4_)_2_SO_4_, containing 3 % glucose, 1 % bacteriological agar (Oxoid)), respectively supplemented with 50 µM or 0.5 mM (NH_4_)_2_SO_4_. Pseudohyphal and growth tests on limiting potassium concentrations were performed on a home-made medium (183) equivalent to Yeast Nitrogen Base medium without amino acids, (NH_4_)_2_SO_4_ and potassium salts, and containing low NaH_2_PO_4_ concentrations. (NH_4_)_2_SO_4_ and KCl were added as required by the experiment and as specified in the text. For growth tests, diploid cells were streaked on SLAD, SHAD and 183 media to follow the formation of colonies.

All growth experiments were repeated at least twice.

***Photomicroscopy of filamentation***

Pictures of yeast colonies were taken directly from Petri plates using a Zeiss Axio Observer Z1 microscope, driven by MetaMorph (MDS Analytical Technologies), with a 10x primary objective and a 2.5x camera adaptor.

***Fluorescence microscopy***

Images of yeast cells were acquired using a Zeiss LSM710 laser-scanning confocal microscope, equipped with the Airy scan module. Acquisitions were performed using the ZEN 2.1 software and images were processed using ImageJ (Figure S3).

**References**

1. Miroux B, Walker JE. 1996. Over-production of proteins in *Escherichia coli*: mutant hosts that allow synthesis of some membrane proteins and globular proteins at high levels. J Mol Biol 260:289-98.

2. Zheng L, Kostrewa D, BernŠche S, Winkler FK, Li XD. 2004. The mechanism of ammonia transport based on the crystal structure of AmtB of *E. coli* ProcNatlAcadSciUSA 101:17090-17095.

3. Mirandela GD, Tamburrino G, Hoskisson PA, Zachariae U, Javelle A. 2018. The lipid environment determines the activity of the *Escherichia coli* ammonium transporter AmtB. FASEB J 33:1989-1999.

4. Bazzone A, Barthmes M, Fendler K. 2017. SSM-Based Electrophysiology for Transporter Research. Methods Enzymol 594:31-83.

5. Gimeno CJ, Ljungdahl PO, Styles CA, Fink GR. 1992. Unipolar cell divisions in the yeast S. cerevisiae lead to filamentous growth: regulation by starvation and RAS. Cell 68:1077-90.
