# Supplementary material for: Coexistence of Ammonium Transporter and Channel Mechanisms in Amt-Mep-Rh Twin-His Variants Impairs the Filamentation Signaling Capacity of Fungal Mep2 Transceptors
Source: mBio. 2022 Mar 1;13(2):e02913-21. doi: 10.1128/mbio.02913-21 (PMC9040831; doi:10.1128/mbio.02913-21)
Supplement: FIG S1 [file mbio.02913-21-sf001.docx]

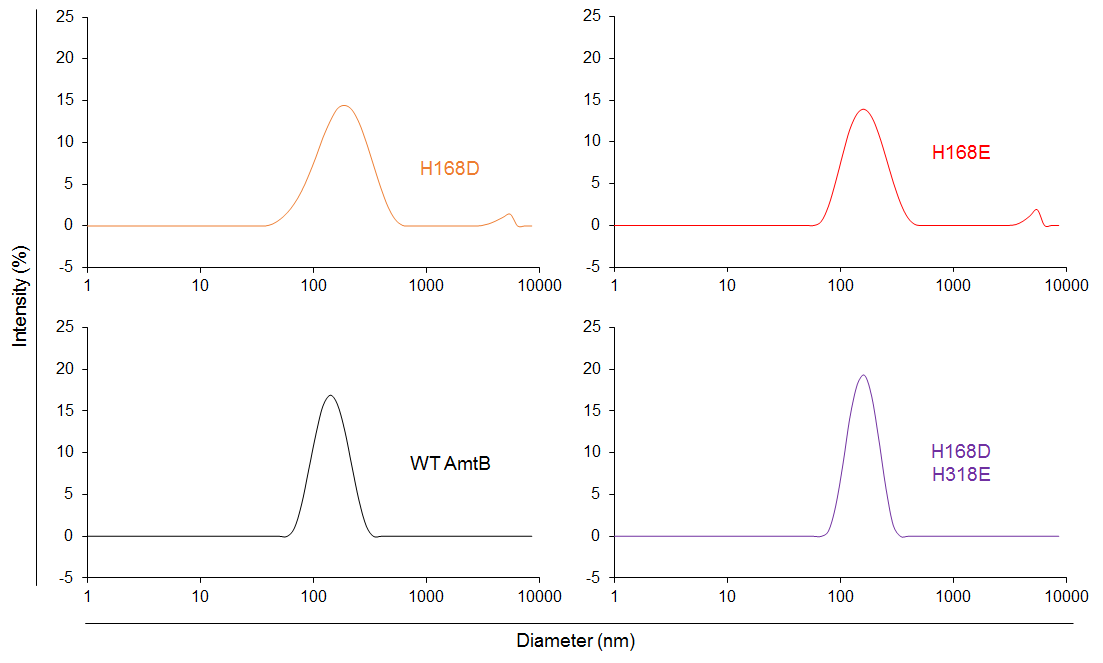


###### **Figure S1: Size distribution of the proteoliposomes containing wild-type and variants of AmtB.** Dynamic light scattering was used to determine the number-weighted distribution of liposome sizes in the detection reagent. The distribution was monodisperse, with a mean diameter of 110 nm.
