# Supplementary material for: Coexistence of Ammonium Transporter and Channel Mechanisms in Amt-Mep-Rh Twin-His Variants Impairs the Filamentation Signaling Capacity of Fungal Mep2 Transceptors
Source: mBio. 2022 Mar 1;13(2):e02913-21. doi: 10.1128/mbio.02913-21 (PMC9040831; doi:10.1128/mbio.02913-21)
Supplement: FIG S2 [file mbio.02913-21-sf002.docx]

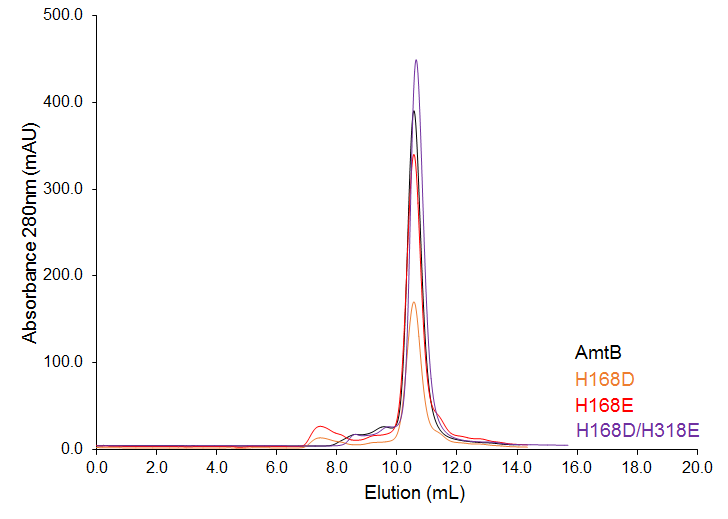


**Figure S2: Size exclusion chromatography analysis of AmtB variants.** Gel filtration trace (Superdex 200 10/300 increase) of wild-type AmtB and variants after solubilization of the proteoliposome in 2% DDM. All of the AmtB variants elute at ~11.5 ml.
