# Supplementary material for: Coexistence of Ammonium Transporter and Channel Mechanisms in Amt-Mep-Rh Twin-His Variants Impairs the Filamentation Signaling Capacity of Fungal Mep2 Transceptors
Source: mBio. 2022 Mar 1;13(2):e02913-21. doi: 10.1128/mbio.02913-21 (PMC9040831; doi:10.1128/mbio.02913-21)
Supplement: TABLE S2 [file mbio.02913-21-st002.docx]

**Table S2: Solid-supported membrane electrophysiology solutions**

| **Substrate** | **Activating** | **Non-Activating** |
| --- | --- | --- |
| Ammonium/MeA | 100 mM KPho  100 mM KCl  200 mM NH_4_Cl/MeACl | 100 mM KPho  300 mM KCl |
| Potassium | 100 mM NaPho  100 mM NaCl  200 mM KCl | 100 mM NaPho  300 mM NaCl |

*Unless otherwise stated, all solutions were adjusted to pH 7. KPho: potassium phosphate buffer, NaPho: sodium phosphate buffer. MeACl: methylammonium chloride. For D_2_O experiments, all solutions were prepared using D_2_O in place of water.
